# Functional Characterization of a Putative *Glycine max ELF4* in Transgenic Arabidopsis and Its Role during Flowering Control

**DOI:** 10.3389/fpls.2017.00618

**Published:** 2017-04-20

**Authors:** Juliana Marcolino-Gomes, Thiago J. Nakayama, Hugo B. C. Molinari, Marcos F. Basso, Liliane M. M. Henning, Renata Fuganti-Pagliarini, Frank G. Harmon, Alexandre L. Nepomuceno

**Affiliations:** ^1^Embrapa Soybean, Brazilian Agricultural Research CorporationLondrina, Brazil; ^2^Embrapa Agroenergy, Brazilian Agricultural Research CorporationBrasília, Brazil; ^3^Plant Gene Expression Center, Agricultural Research Service – United States Department of Agriculture, AlbanyNY, USA; ^4^Department of Plant and Microbial Biology, University of California-Berkeley, BerkeleyCA, USA

**Keywords:** *A. thaliana*, circadian clock, flowering time, gene expression, overexpression, soybean, transgenic

## Abstract

Flowering is an important trait in major crops like soybean due to its direct relation to grain production. The circadian clock mediates the perception of seasonal changes in day length and temperature to modulate flowering time. The circadian clock gene *EARLY FLOWERING 4* (*ELF4*) was identified in *Arabidopsis thaliana* and is believed to play a key role in the integration of photoperiod, circadian regulation, and flowering. The molecular circuitry that comprises the circadian clock and flowering control in soybeans is just beginning to be understood. To date, insufficient information regarding the soybean negative flowering regulators exist, and the biological function of the soybean *ELF4* (*GmELF4*) remains unknown. Here, we investigate the *ELF4* family members in soybean and functionally characterize a *GmELF4* homologous gene. The constitutive overexpression of *GmELF4* delayed flowering in Arabidopsis, showing the *ELF4* functional conservation among plants as part of the flowering control machinery. We also show that *GmELF4* alters the expression of Arabidopsis key flowering time genes (*AtCO* and *AtFT*), and this down-regulation is the likely cause of flowering delay phenotypes. Furthermore, we identified the *GmELF4* network genes to infer the participation of *GmELF4* in soybeans. The data generated in this study provide original insights for comprehending the role of the soybean circadian clock ELF4 gene as a negative flowering controller.

## Introduction

Soybean (*Glycine max* L. Merrill) is a globally important leguminous crop that produces high-quality protein and oil used in human and animal feed and has potential for biofuels. Sustainable intensification of crop production is crucial for meeting the food demand of the growing human population (Godfray et al., 2010). Flowering is directly related to seed production and, thus, to grain productivity. Hence, understanding the genes and the molecular network that control flowering is of great interest to improve crop productivity.

The circadian clock is a molecular oscillator that controls fundamental aspects of plant development and metabolism, from gene expression and protein catabolism to control of leaf position and flowering (McClung, 2011). It is believed to enhance fitness by synchronizing the metabolic and physiological processes to the different periods of the day and is considered an important adaptive characteristic (Yerushalmi and Green, 2009). The plant circadian clock interprets seasonal changes in day length and temperature in order to modulate flowering time, ensuring that the transition from vegetative to reproductive stages occurs under optimal physiological and environmental conditions, enhancing reproductive success (Adams and Carré, 2011).

The most complete existing model of the circadian clock in plants is described for Arabidopsis (*Arabidopsis thaliana*) and is composed of interlocked feedback loops, which includes a loop of three sequential negative steps: (a) the inhibition of evening complex (EC) genes (*ELF3, LUX*, and *ELF4*) (Nusinow et al., 2011) by the increase of LHY/CCA1 during the late night, (b) the inhibition of PRR genes by the EC during the early night, and (c) the inhibition of LHY/CCA1 by PRRs during the day (Pokhilko et al., 2012). In this model, the two partially redundant morning MYB-like transcription factors CCA1 and LHY reach peak levels in the morning to repress the daytime expression of *TOC1* and *GI* (Harmer et al., 2000; Alabadí et al., 2001). It was also demonstrated that the inhibitory action of LHY/CCA1 extends to all evening genes (*TOC1, LUX, ELF4, ELF3*, and *GI*) (Pokhilko et al., 2012). In turn, TOC1 appears to repress *CCA1* and *LHY* expression within the morning loop (Gendron et al., 2012; Pokhilko et al., 2012), while the EC indirectly activates *CCA1*/*LHY* expression by suppressing the expression of *CCA1*/*LHY*’s repressors (Kikis et al., 2005; Dixon et al., 2011; Helfer et al., 2011; Pokhilko et al., 2012). On the other hand, GI increases *TOC1* expression by the inhibition of the EC, which is a negative regulator of *TOC1* expression (Pokhilko et al., 2012). Together, the three interlocking feedback loops ensure that the clock produces robust and accurate rhythms necessary to control plant physiological and developmental processes, among them, flowering time.

The photoperiodic-dependent regulation of flowering also has been covered in detail in Arabidopsis, a facultative long-day plant [the floral transition occurs earlier when plants are grown under long days (LD) than when they are grown under short days (SD)]. In this plant, the perception of day length and flowering is mainly coordinated by the transcription factor CONSTANS (CO), and the expression of *CO* is finely tuned by the circadian clock (Wenkel et al., 2006). Under SD conditions, the expression of the *CO* mRNA occurs after dark, with RNA levels peaking 16 h after dawn, and the CO protein is rapidly degraded by the action of COP1 and the proteasome, resulting in flowering inhibition (Jang et al., 2008). Conversely, under LD conditions, the peak of *CO* expression occurs during daylight, when the activity of COP1 is inhibited, resulting in CO protein accumulation, and, consequently, flowering induction by the activation of the floral integrators *FLOWERING TIME* (*FT*) and *TWIN SISTER OF FT* (*TSF*) (Yamaguchi et al., 2005; Jang et al., 2008). In addition, the F-box protein and blue light photoreceptor FKF1 and GI act synergistically to promote the degradation of CDF1, a transcriptional repressor, enabling *CO* expression at the end of a long day (Sawa et al., 2007). The product of *FT* expression migrates to the apical meristem, where it initiates the transcriptional reprogramming that results in flowering (Turnbull, 2011).

The circadian clock gene *EARLY FLOWERING 4* (*ELF4*) is believed to play a key role in the integration of photoperiod perception, circadian regulation, and flowering (Doyle et al., 2002). The *ELF4* gene was identified through reverse genetic screens for flowering time mutants. The *elf4* mutants have elongated hypocotyls and petioles, flower early and lack the ability to sense day length. The early flowering behavior of *elf4* mutants is accompanied by misregulation of the flowering activator CO (Doyle et al., 2002). Molecular analysis data reveal ELF4 sequesters GI from the nucleoplasm by a physical interaction, impeding GI to bind at the promoter of *CO*, altering flowering time (Kim et al., 2013).

*ELF4* is essential for the maintenance of circadian rhythms, since the *elf4* mutation disrupts rhythmic *CCA1* expression (Doyle et al., 2002). It is also known that *ELF4*, together with *TOC1*, plays a major role in phytochrome B-mediated input to the clock (red light perception) (Kikis et al., 2005). The Arabidopsis ELF4 protein has 111 amino acids and does not contain any known protein signatures (Doyle et al., 2002). This protein belongs to the DUF1313 family, which is a family of proteins that share a domain of unknown function exclusively found in plants (DUF1313).

In contrast to the large volume of data on flowering in LD plants, in SD plants, such as rice and soybeans, the molecular basis of flowering control is still being identified and characterized. Most soybean cultivars have a SD requirement for floral induction: flowering remains suppressed under LD conditions and is induced when the day length is shorter than a critical length, and this sensitivity to photoperiod varies among cultivars (cultivars adapted to high latitudes have weak or absent sensibility to photoperiodic changes) (Watanabe et al., 2012).

There are at least 10 *FT* genes in soybean, arranged in five sets of gene pairs linked in tandem (Kong et al., 2010). Many studies on the photoperiod response indicate that *FT* and its homologes are universal signaling molecules for flowering plants (Turnbull, 2011). Furthermore, 26 genes orthologous to the *CO* gene of Arabidopsis were identified in soybean, named *G. max CONSTANS*-*Like* (*GmCOL*). Gene expression analysis revealed that in SD conditions *GmCOL1a* and *GmCOL1b* mRNA peaks occur at the end of the morning and coincide with the peak of the soybean *FT* orthologous gene, *GmFT5a*, inducing flowering. On the other hand, under LD, the expression of *GmCOL1a* and *GmCOL1b* occurs during the night and declines before dawn, and in those conditions the expression of *GmFT5a* is not induced (Wu et al., 2014).

Although some information about soybean flowering induction genes (*FT* and *CO*) exists, at the moment, there is no information regarding soybean negative flowering regulators, such as *ELF4*. In a previous study, we identified a GmELF4 candidate gene and showed its daily expression pattern of mRNA (Marcolino-Gomes et al., 2014). In this context, here we identify and characterize soybean *ELF4* genes and show how a *GmELF4* homolog alters the expression of key flowering genes in transgenic Arabidopsis, resulting in flowering delay.

## Materials and Methods

### Identification, Phylogenetic Relationships, and Protein Domain Analysis of ELF4 Members in Soybean and Other Plants

For the identification of putative soybean *ELF4* genes and proteins, the corresponding Arabidopsis ELF4 protein sequence (encoded by AT2G40080) was used as a query in BLAST searches (TBLASTN tool) (Altschul et al., 1990) of the *G. max* genome version Wm82.a2.v1, available from the Phytozome database^1^, using a cutoff Expect value (E-value) of 1E-12. The soybean *ELF4* genes and proteins are representative of the respective gene model ‘Glymas.’ The ELF4 homologous proteins in plant species were searched for in the NCBI protein database^2^, using keyword searches and are represented by their respective GenInfo Identifier number (GI number).

Phylogenetic relationships between the soybean and other plant ELF4 proteins were considered. For this purpose, after removing the redundancy, the amino acid sequences were subjected to global alignment using the ClustalW algorithm from the Molecular Evolutionary Genetics Analysis version 5.0 (MEGA 5) software package (Tamura et al., 2011), and a phylogenetic tree was constructed using the neighbor-joining (NJ) method with the following parameters: Poisson correction, pairwise deletion and bootstrap (1000 replicates; random seed). Given that the functional domains have importance for the function and classification of ELF4 proteins, the conserved amino acid domains of the ELF4 proteins were analyzed using the motif discovery tool MEME program version 4.11.2^3^.

### *ELF4* Soybean Paralogous Genes’ Expression

In a previous study, we identified a *GmELF4* candidate gene Glyma.18G027500 (former Glyma18g03130) and showed its daily expression pattern of mRNA using RT-qPCR (Marcolino-Gomes et al., 2014). In the present study, the gene expression patterns of all *ELF4* soybean paralogous genes were assessed through analysis of an RNA-Seq database^4^, built from soybean plants of the BR16 genotype (Rodrigues et al., 2015). Plants were grown in 14 h light/10 h dark cycles, with 500 μmol m^-2^ s^-1^ of white light (provided by cool white fluorescent bulbs), under 28°C/20°C temperature cycles during the light and the dark periods, respectively. Sampling was performed 15 days after germination, when the plants reached the V2 developmental stage (Fehr et al., 1971), at which fully expanded V1 leaves were collected from the three plants at 4-h intervals from the time the lights came on [Zeitgeiber time (ZT) 0].

Total RNA was extracted from leaves using the RNA Plant Reagent^®^ according to the manufacturer’s instructions (Ambion, Austin, TX, USA) and treated with DNAse (Invitrogen, Carlsbad, CA, USA). High-quality total RNA (RIN ≥ 8.0) was used to analyze the transcripts for each time point: ZT0, 4, 8, 12, 16, and 20. Equimolar quantities of purified total RNA samples from two independent biological replicates were pooled into one template for library synthesis. For each time period/treatment, three independent libraries were synthesized. The RNA-Seq libraries were built using the NuGEN OvationH kit according to the manufacturer’s instructions (NuGEN Technologies, Inc., San Carlos, CA, USA). The libraries obtained were subjected to sequencing by an Illumina HiSeq 2000 system (Illumina, San Diego, CA, USA), using the paired-end mode. Mapping of the reads was performed with the soybean genome (Phytozome *Glycine max* version 1.1) using the GeneSifter platform^5^. To compare gene expression profiles between different times and treatments (control and drought stress), we log_2_-transformed the normalized reads [mapped reads per million (RPM)]. The gene expression data obtained at the RNA-seq analyses was validated by qPCR (Rodrigues et al., 2015).

### Overexpression of *GmELF4* in Arabidopsis

The coding sequence (CDS) of the *GmELF4* gene (Glyma.18G027500) was amplified from genomic DNA from soybean (genotype Williams 82) by PCR reactions using the specific 5′-CACCATGGAAGACCCCTC-3′ and 5′-TTATTTGGAGGAGTTTTTGGAG-3′ forward and reverse primers, respectively, and the Platinum^®^ Taq DNA Polymerase High Fidelity (ThermoFisher Scientific, Waltham, MA, USA). The sequence 5′-CACC-3′ was added to the 5′ upstream region of the forward primer to ensure directional subcloning of the CDS with the pENTR^TM^/D-TOPO^®^ (ThermoFisher Scientific, Waltham, MA, USA) entry vector. After confirmation by sequencing, the *GmELF4* CDS was transferred to the destination overexpression vector p^∗^7WG2D (Karimi et al., 2002) using the Invitrogen^TM^ Gateway^TM^ recombination cloning system (ThermoFisher Scientific, Waltham, MA, USA). With the p^∗^7WG2D binary vector, the *GmELF4* CDS is constitutively expressed due the effect of the *Cauliflower mosaic virus 35S* (*CaMV35S*) promoter. In addition, the construct cassette harbors a rolD promoter fused to the CDS of the enhanced green-fluorescent protein (GFP) linked to an endoplasmic reticulum-targeting signal (pRolD-EgfpER-T35S), used as a fluorescence marker (Karimi et al., 2002).

The resultant gene construct was used for *Arabidopsis thaliana* ecotype Columbia (*Col-0*) transformation mediated by *Agrobacterium tumefaciens* (strain GV 3101) using the floral dip method (Zhang et al., 2006). Seeds were screened in 1% agar containing Murashige and Skoog (MS) medium containing 15 μg mL^-1^ hygromycin B, as described by Harrison et al. (2006). Hygromycin-resistant plants were confirmed via PCR using specific primers for the gene construct (described above). Since the selection of transgenic lines with low T-DNA copy number is advised to prevent transgene silencing, in our study, we carefully evaluated the T-DNA segregation during three generations, selecting lines that presented Mendelian inheritance for a single characteristic [three transformed plants: one wild-type (WT); 3:1], which would carry just one copy of the transgene. Three independent T3 homozygous lines containing a single copy of the transgene were obtained for further analysis.

### Transgenic Plants Phenotype

The development of plants from three transgenic lines was monitored in growth chambers with controlled temperature (22 ± 2°C) and relative humidity (50 ± 10%). Plants were cultivated under LD and SD conditions, with 16 and 10 h of light, respectively. After plant emergence, the rosette leaf numbers, flowering time and progression of silique formation were monitored weekly. Each line was evaluated via 10 biological replicates. Transgenic plants developmental features were compared to those of non-transformed plants (WT) in both photoperiodic conditions.

### GFP Detection in Transgenic Plants

Green-fluorescent protein fluorescence was evaluated in leaves and roots of 5-day-old plants. Fresh tissue was analyzed with an Axio Scope A1 microscope (Zeiss, Oberkochen, Germany) equipped with a GFP filter (Zeiss filter set 38; Zeiss) coupled to a charge-coupled device (CCD) camera (Motic, Causeway Bay, Hong Kong) for image acquisition Image was captured using Motic Image Plus version 2.0 software (Motic, Causeway Bay, Hong Kong) with the following parameters: exposure = 500, gain = 2.38, gamma = 0.10/191 and size = 2592 × 1944 pixels. The image was magnified by a factor of 100x, which corresponds to the product of the objective magnification (10x) and the eyepiece lens (10x).

Image analysis for GFP quantitation was performed with ImageJ software version 1.51 h^6^. In this analysis, images were converted to 32-bit type and the mean gray values were measured using the ROI analysis tool. The mean gray values of WT plants were used for background normalization of GFP intensity in transgenic plants, generating the relative intensity values. A total of 30 independent sample areas were analyzed in each image.

### Gene Expression Analysis via RT-qPCR

The overexpression of *GmELF4* (Glyma.18G027500) and the expression of the Arabidopsis flowering genes *AtFT* (AT1G65480) and *AtCO* (AT5G15840) were evaluated in transgenic Arabidopsis via RT-qPCR assays.

Primers for *GmELF4* (forward 5′-TGATTCAGCAGGTGAACGAG-3′ and reverse 5′-GACAACCTTGGAGATGTTGC-3′) were designed with the Primer3 Plus online tool (Rozen and Skaletsky, 1999)^7^, based on the Glyma.18G027500.1 transcript sequence^8^. The Arabidopsis *AtCO* and *AtFT* primer sequences were retrieved from Ito et al. (2012).

Gene expression was evaluated in rosette leaves of three independent transgenic lines and in WT plants, cultivated in LD and SD conditions, with 16 and 10 h of light, respectively. For RNA extraction, each replicate tissue was ground to a fine powder in liquid nitrogen, and total RNA was isolated using TRIzol reagent (Invitrogen), according to the manufacturer’s instructions. Contaminating DNA in the total RNA was removed using a TURBO DNA-free Kit, according to the manufacturer’s instructions (Life Technologies, Grand Island, NY, USA). High-quality total mRNA was used to synthesize cDNA strands (Superscript III First Strand Synthesis, Invitrogen/Life Technologies, Grand Island, NY, USA). The quality of the cDNA and contamination with genomic DNA were examined using a standard PCR assay with primers that spanned an intronic region of the *β-ACTIN* Arabidopsis gene (AT5G09810). High-quality cDNA was used to analyze the transcripts in each treatment.

RT-qPCR amplifications were performed in a 7300 RT-qPCR Thermocycler (Applied Biosystems/Life Technologies, Grand Island, NY, USA) with the following cycling parameters: 50°C for 2 min; 95°C for 10 min; and 45 cycles of 95°C for 2 min, 60°C for 30 s and 72°C for 30 s. Standard curves were produced from serial dilutions of a cDNA pool to estimate the efficiency of the PCR amplification with each pair of primers. The primer concentrations were adjusted to achieve efficiency rates higher than 85%.

After carrying out the efficiency analysis, the expression levels of the transgene (*GmELF4*) and flowering genes (*AtFT* and *AtCO*) were analyzed separately in transgenic and WT plants, cultivated in LD and SD conditions. The reactions were performed in triplicate, with cycling parameters similar to those described above for the amplification efficiency analysis. Expression of target genes was normalized using the Arabidopsis endogenous gene *PP2A* (AT1G13320) (forward primer 5′-ACGTGGCCAAAATGATGCAA-3′ and reverse primer 5′-TCATGTTCTCCACAACCGCT-3′). The flowering genes (*AtFT* and *AtCO*) relative expression was calibrated using WT plants grown under the same environmental conditions. The gene expression analysis was performed using the Rest2009 software package version 2.0.13 (Pfaffl et al., 2002).

### The *GmELF4* Network Genes

The *GmELF4* gene co-expression regulatory network was evaluated with the Soybean Functional Genomics Database (SFGD) platform (Yu et al., 2014). The SFGD database contains microarray gene expression profiles of 255 samples from experiments of 14 groups and mRNA-Seq data of 30 samples from experiments of four groups, including spatial and temporal transcriptome data for different soybean developmental stages and environmental stresses, providing a robust database for functional analysis of genes of interest. This platform is publically accessible^9^. Pearson’s correlation coefficients (PCCs) and mutual rank (MR) for gene co-expression (Obayashi and Kinoshita, 2009) were used to construct the *GmELF4* network. A table including the top 50 genes with the greatest MR values was constructed. The Cytoscape Web software program version 3.4.0 (Lopes et al., 2011) was used to display the *GmELF4* (central node) and all its co-expression genes, sorted according to MR values.

## Results

### Identification and Characterization of *ELF4* Genes in Soybean

**FIGURE 1:**
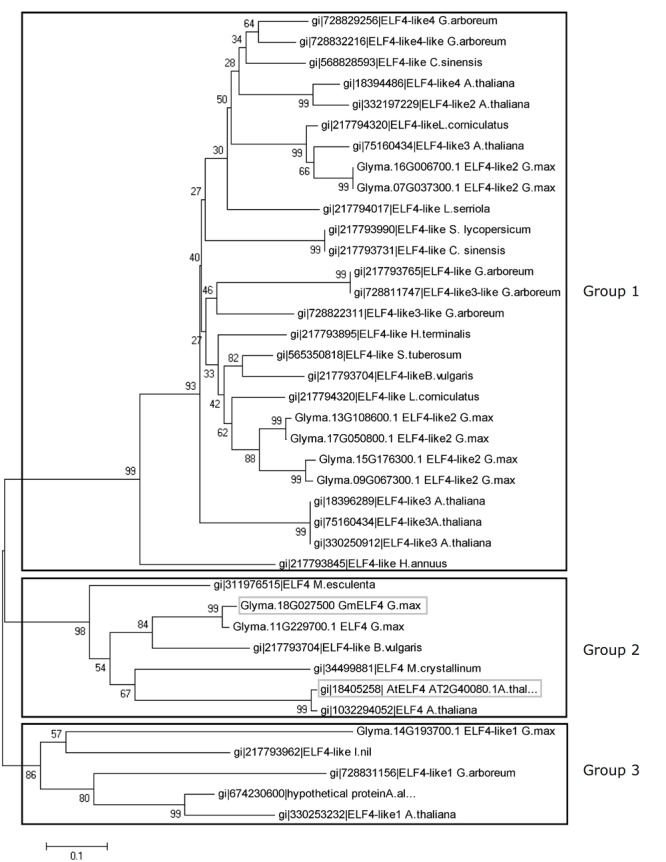
**Evolutionary relationships of plant ELF4.** The evolutionary history of 39 ELF4 plant proteins was inferred using the neighbor-joining method. The bootstrap consensus tree inferred from 1000 replicates is taken to represent the evolutionary history of the taxa analyzed. Branches corresponding to partitions reproduced in less than 50% of bootstrap replicates are collapsed. The percentage of replicate trees in which the associated taxa clustered together in the bootstrap test (1000 replicates) is shown next to the branches. The evolutionary distances were computed using the Poisson correction method and are in units of numbers of amino acid substitutions per site. All positions containing alignment gaps and missing data were eliminated only in pairwise sequence comparisons (pairwise deletion option). There were a total of 212 positions in the final dataset. Phylogenetic analyses were conducted with MEGA5 software (Tamura et al., 2011). The soybean *ELF4* candidate gene (Glyma.18G027500) and the Arabidopsis *ELF4* gene (AT2G40080) are highlighted in gray boxes.

In this study, the soybean *ELF4* candidate genes were identified using Arabidopsis *ELF4* coding-sequences for *in silico* searches of the soybean genome. Nine *GmELF4*-like genes were identified (Glyma.07G037300, Glyma.09G067300, Glyma.18G027500, Glyma.11G229700, Glyma.13G108600, Glyma.14G193700, Glyma.15G176300, Glyma.16G006700, and Glyma.17G050800). To establish the relationships between the soybean ELF4 candidates and ELF4 proteins of Arabidopsis and other plants, we performed global alignments, followed by phylogenetic analysis and construction of a phylogenetic tree (**Figure 1**). This tree indicates three large groups of ELF4 proteins. The soybean proteins encoded by *GmELF4-like2* genes are gathered into group 1 with the Arabidopsis ELF4-like2, ELF4-like3, and ELF-like4 as well as with ELF4-like proteins from several other organisms. In group 2, the soybean ELF4 proteins encoded by Glyma.11G229700 and Glyma.18G027500 show close relation to the Arabidopsis ELF4 major protein (AT2G40080) (**Figure 1**). Group 3 constitutes the ELF4-like1 proteins from Arabidopsis, soybean and other plants (**Figure 1**).

**FIGURE 2:**
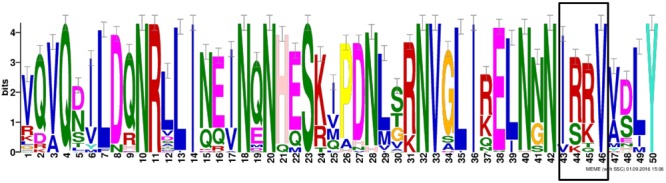
**Conserved amino acid motifs of the DUF1313 domain in ELF4 proteins in plants.** Abscissa indicates amino acid residue number, and vertical axis indicates residue height, as described by the percentage of residues in the type. The box indicates the four conserved amino acid residues of the IRRV-type proteins from the DUF1313 family.

Considering the importance of the functional domain for the classification and for investigating more deeply the putative function of the soybean candidates of *ELF4*, we performed a motif analysis of nine soybean ELF4 proteins together with 30 other plant ELF4 proteins (**Figure 2**). Our analysis revealed the presence of a DUF1313 motif conserved in all 39 ELF4 proteins, including the soybean *ELF4* candidate genes Glyma.11G229700 and Glyma.18G027500. The Arabidopsis ELF 4 belongs to the DUF1313 family, whose members are divided into three major types, according to the substitution of four amino acid residues: IARV type, I(S/T/F)(K/R)V type and IRRV type, where the IRRV type is the largest (158 genes) (Li et al., 2015). Our analysis shows that the *GmELF4* putative genes contain residues belonging to the IRRV type genes (**Figure 2**).

### *GmELF4* Gene Expression in Soybeans

**FIGURE 3:**
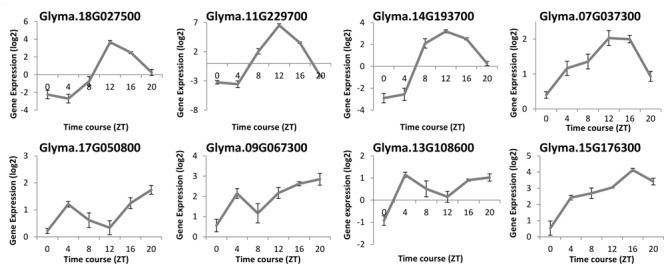
**RNA-Seq data of the soybean *ELF4* homologous genes.** Gene expression was measured in leaf tissues of BR16 soybeans subjected to short day (SD) conditions. Gene expression (Exp. Log. [log_2_]) was evaluated over a 24-h time course from the time the light came on (ZT0), in 4-h intervals. Error bars represent standard error (SEM).

To compare the diurnal oscillation of gene expression of *ELF4* in soybeans and Arabidopsis, we evaluated the transcription levels of eight *GmELF4* homologous genes by re-analyzing a validated RNA-Seq library published by Rodrigues et al. (2015). The expression profiles of the soybean *ELF4* candidates were determined in V2-stage soybean plants under LD conditions (14 h light/10 h dark) and temperature (28°C/20°C) cycles over a 24-h time course under well-watered conditions (**Figure 3**).

The mRNA levels of Glyma.18G027500, Glyma.11G229700, and Glyma.14G193700 peak at ZT12. The peak of transcripts of Glyma.07G037300 also occurs at ZT12, but, differently from the former genes, this peak is extended to ZT16. In contrast, Glyma.17G050800, Glyma.13G108600, and Glyma.09G067300 show two peaks of transcripts: one at ZT4 and another at ZT20. For instance, the Glyma.15G176300 expression level rises gradually throughout the day, peaking at ZT16 (**Figure 3**).

### Functional Characterization of a Soybean Putative *ELF4* Homologous Gene by Overexpression in Arabidopsis Plants

To functionally characterize a soybean *ELF4* homolog (*GmELF4*), we transformed Arabidopsis plants to overexpress constitutively the CDS of Glyma.18G027500. This soybean *ELF4* homologous gene was selected due its resemblance to the features of *AtELF4* at the phylogenetic and transcriptomic levels: the protein sequence of Glyma.18G027500 is very closely related to that of *AtELF4* (**Figure 1**), and its transcripts oscillate throughout the day with the same profile of the *AtELF4* transcripts (**Figure 3**).

#### Molecular Characterization of Transgene Expression in Arabidopsis *GmELF4-ox*

**FIGURE 4:**
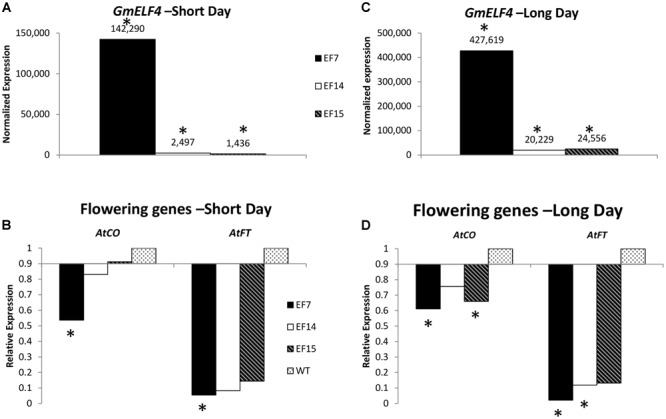
**Gene expression profile by RT-qPCR.** The expression of *GmELF4* **(A)** and of the flowering genes *AtFT* and *AtCO* **(B)** was evaluated in under short conditions. **(C,D)** Show the expression of *GmELF4* and the flowering genes (*AtFT* and *AtCO*) under long day conditions. Leaves of the transgenic lines (EF7, EF14, and EF15) were evaluated at ZT8. Relative expression was calculated using the Rest2009 software package (Pfaffl et al., 2002) using the Arabidopsis endogenous gene *PP2A* for expression normalization. Wild-type (WT) plants expression was used for *AtFT* and *AtCO* expression calibration **(B,D)**. Asterisks indicate statistically significant changes in gene expression, as calculated by Rest2009.

To evaluate the expression of *GmELF4* in the three independent *GmELF4*-ox lines (EF7, EF14, and EF15), we analyzed the transcript levels of *GmELF4* in leaves of transgenic versus WT plants cultivated under LD and SD conditions (**Figures 4A,C**). Our gene expression data show that *GmELF4* was highly expressed in line EF7 for both SD and LD, while lines EF14 and EF15 had lower transcript levels (**Figures 4A,C**). The transgene expression was also detected by GFP fluorescence emission in leaves and roots of transgenic lines (Supplementary Figure 1).

#### Transgenic Plants Phenotypes

**FIGURE 5:**
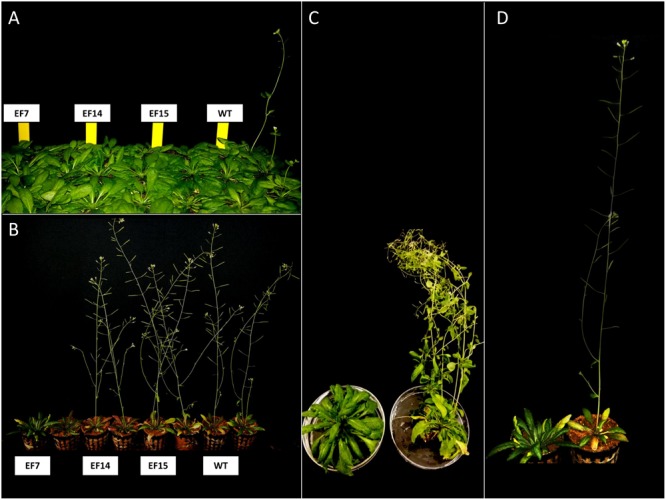
**Flowering in transgenic Arabidopsis plants.** Transgenic (lines EF7, EF14, and EF15) and WT plants were grown under short-day (SD) and long-day (LD) with 10 and 16 h of light, respectively. Representatives of the earliest and latest flowering plants are shown for each genotype: **(A)** 8 week-old plants cultivated under SD; **(B)** 6 week-old plants cultivated under LD conditions. The contrasting flowering phenotypes of transgenic line EF7 and WT grown under SD **(C)** and LD **(D)** conditions are shown in detail: **(C)** 12 week-old plants cultivated under SD; **(D)** 6 week-old plants cultivated under LD.

The genetic transformation of the three independent lines overexpressing the putative *GmELF4* (*GmELF4*-ox), named EF7, EF14 and EF15, was confirmed by PCR using primers specific to the *GmELF4* CDS (Supplementary Figure 2). The most striking phenotype of the transgenic *GmELF4*-ox lines compared to WT plants was flowering delay. Transformed Arabidopsis plants cultivated in both flowering induction conditions (LD) and non-inductive conditions (SD) displayed a later transition from the vegetative to reproductive developmental phase (**Figure 5** and Supplementary Figures 3, 4). Interestingly, the EF7 line displayed the most delayed flowering phenotype in response to both photoperiods, while WT plants were the first to flower (**Figure 5** and Supplementary Figures 3, 4).

**FIGURE 6:**
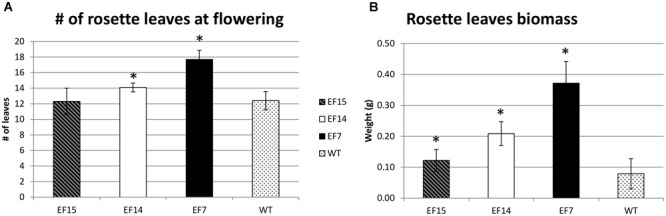
**Rosette leaves during development.** The number of rosette leaves at beginning of flower **(A)**, and the Rosette leaves biomass **(B)** are presented for transgenic lines (EF7, EF14, and EF15) and WT plants cultivated under 16 h of light (LD). The rosette leaf numbers were recorded when the primary inflorescence stem reach to 1 cm. Mean values from 10 biological replicates are presented; error bars represent the standard deviation. Asterisks indicate statistical differences between the transgenic lines and WT values (Student’s *t*-test *p* < 0.05).

Another interesting feature of transgenic plants was the higher number of rosette leaves compared to WT plants, especially in transgenic line EF7 (**Figure 6**). This transgenic line had more leaves at the onset of the developmental transition from the vegetative to reproductive phase (**Figure 6A**), resulting in higher biomass accumulation (**Figure 6B**).

#### Molecular Characterization of Key Flowering Genes in *GmELF4-ox* Arabidopsis

The notable flowering delay of *GmELF4*-ox Arabidopsis encouraged us to investigate the molecular mechanisms behind the observed phenotypes.

To evaluate the impact of the overexpression of the soybean *ELF4* homologous gene (Glyma.18G027500) on the expression of the key Arabidopsis flowering genes *AtFT* and *AtCO*, we analyzed transcript levels in transgenic plants compared to WT plants. Interestingly, our data reveal that the overexpression of *GmELF4* had a significant inverse effect of impact on the levels of *AtFT* and *AtCO* transcripts, especially in the EF7 line, where the high levels of *GmELF4* (**Figures 4A,C**) caused a substantial reduction in expression of *AtFT* and *AtCO* (**Figures 4B,D**). Similarly, lines EF14 and EF15 also presented reduced levels of *AtFT* and *AtCO* compared to WT plants, albeit in a slighter manner (**Figures 4B,D**).

### The Role of *GmELF4* in Soybeans: Network Genes

To better understand the function of the putative *GmELF4* (Glyma.18G027500) in soybeans, we retrieved functionally related genes by constructing a co-expressed gene network using the SFGD database. The top 50 co-expressed genes from the *GmELF4* network are listed in **Table 1**. A graphical representation of this network (Supplementary Figure 5) shows *GmELF4* at the core of a network composed of genes that connect directly or indirectly to the core gene. Among those genes are several homologes of the circadian clock genes, including the *GmELF4* paralog Glyma.11G229700, *JUMONJI* (Glyma.12G055000), *CO-like* (Glyma.02G223700, Glyma.16G050900, and Glyma.14G190400), and *EARLY FLOWERING 3* (Glyma.04G050200) as well as the gene encoding *GIGANTEA* (GI) (Glyma.16G163200) (**Table 1**).

**Table 1:** Top 50 *GmELF4* network genes.

MR^1^	PCC^2^	*G. max* v1.0^3^	*G. max* Wm82.a2.v1^4^	Annotation^5^
1.0	0.899	Glyma11g35270	Glyma.11G229700	EARLY FLOWERING 4-like
5.5	0.729	Glyma12g05890	Glyma.12G055000	JUMONJI transcription factor
6.9	0.764	Glyma17g36060	Glyma.17G242100	COLD REGULATED PROTEIN 27
7.6	0.768	Glyma20g03910	Glyma.20G031200	Unknown protein
11.6	0.680	Glyma02g38870	Glyma.02G223700	C2C2 (Zn) CO-like transcription factor
12.7	0.624	Glyma18g46020	Glyma.18G226200	METHYLTRANSFERASE
14.4	0.730	Glyma02g45810	Glyma.02G288700	HEAT SHOCK PROTEIN 42
19.6	0.672	Glyma11g19500	No mapping	
20.0	0.694	Glyma09g07240	Glyma.16G163200	PROTEIN GIGANTEA
20.5	0.696	Glyma06g35550	Glyma.06G235100	Protein of unknown function (DUF3082)
21.4	0.693	Glyma14g02970	Glyma.14G026100	HEAT SHOCK PROTEIN 42
23.7	0.520	Glyma04g05280	Glyma.04G050200	Protein EARLY FLOWERING 3
24.3	0.676	Glyma18g20600	Glyma.18G142500	Alpha-glucan, water dikinase/starch-related R1 protein
26.5	0.515	Glyma16g05540	Glyma.16G050900	ZINC FINGER PROTEIN CONSTANS-LIKE 14-RELATED
26.7	0.642	Glyma18g05060	Glyma.18G044300	ANKYRIN REPEAT DOMAIN-CONTAINING PROTEIN EMB506
28.6	0.466	Glyma14g36930	Glyma.14G190400	C2C2 (Zn) CO-like transcription factor
32.0	0.665	Glyma02g15370	Glyma.02G136000	Ent-kaurene synthase/Ent-kaurene synthetase B
36.1	0.604	Glyma05g29200	Glyma.05G159400	SHAGGY-RELATED PROTEIN KINASE ETA
37.1	0.603	Glyma02g45280	Glyma.02G283500	HXXXD-TYPE ACYL-TRANSFERASE-RELATED
37.4	0.494	Glyma20g33810	Glyma.20G196000	GLUCOSYL/GLUCURONOSYL TRANSFERASES
40.5	0.618	Glyma17g12800	No mapping	
46.3	0.640	Glyma07g01400	Glyma.07G011700	PHOSPHATASE, ORPHAN 1, 2
46.4	0.631	Glyma18g07520	Glyma.18G067500	Similar to maternal effect embryo arrest 5
53.6	0.577	Glyma02g46870	Glyma.02G299100	SERINE ACETYLTRANSFERASE 1, CHLOROPLASTIC-RELATED
54.7	0.608	Glyma08g45150	Glyma.08G334500	Similar to maternal effect embryo arrest 5
56.9	0.524	Glyma12g04750	No mapping	
59.8	0.673	Glyma07g31740	Glyma.07G197600	Molecular chaperone (DnaJ superfamily)
60.9	0.572	Glyma17g13780	Glyma.17G128500	MYB/HD-like transcription factor
61.9	0.556	Glyma16g33060	Glyma.16G205500	APO PROTEIN 1, CHLOROPLASTIC
62.4	0.551	Glyma11g15600	Glyma.U034800	Unknown protein
66.3	0.566	Glyma13g40850	Glyma.13G333300	METHYL-CPG-BINDING DOMAIN-CONTAINING PROTEIN 1-RELATED
75.9	0.527	Glyma06g13190	Glyma.06G126500	PROTEIN DHS-1
78.4	0.529	Glyma17g18010	Glyma.17G165200	METAL TRANSPORTER NRAMP2-RELATED
83.0	0.634	Glyma12g15560	Glyma.12G130000	DNAJ HOMOLOG DNJ-5
83.9	0.620	Glyma08g12370	Glyma.08G117200	SHAGGY-RELATED PROTEIN KINASE ETA
85.9	0.671	Glyma16g26900	Glyma.16G153000	NDH-DEPENDENT CYCLIC ELECTRON FLOW 5
86.4	0.456	Glyma06g24420	No mapping	
90.0	0.503	Glyma13g31600	No mapping	
91.6	0.576	Glyma03g03590	Glyma.03G030600	CYTOCHROME P450 71B21-RELATED
93.2	0.573	Glyma06g13450	Glyma.06G129100	ATP-DEPENDENT CLP PROTEASE REGULATORY SUBUNIT CLPX
100	0.466	Glyma18g14490	No mapping	
105	0.541	Glyma06g20830	Glyma.06G193800	GIBBERELLIN-REGULATED PROTEIN 12-RELATED
105	0.620	Glyma11g21670	Glyma.11G155300	Transglutaminase-like superfamily (Transglut_core2)
107	0.559	Glyma03g03630	Glyma.03G030800	CYTOCHROME P450 71B21-RELATED
108	0.655	Glyma08g39110	Glyma.08G283700	Alpha-glucan, water dikinase/starch-related R1 protein
109	0.652	Glyma16g29640	Glyma.16G177800	Protein of unknown function (DUF3464) (DUF3464)
111	0.592	Glyma15g11800	Glyma.15G111400	CHAPERONIN-LIKE RBCX PROTEIN
119	0.479	Glyma18g51380	Glyma.18G278800	*S*-ADENOSYLMETHIONINE DECARBOXYLASE PROENZYME-RELATED
119	0.456	Glyma15g40940	Glyma.15G257600	Deacetoxyvindoline 4-hydroxylase/desacetyoxyvindoline-17-hydroxylase
120	0.438	Glyma08g22370	Glyma.08G209300	Unknown protein

*^1^Mutual rank (MR) for gene co-expression (Obayashi and Kinoshita, 2009);*

*^2^Pearson’s correlation coefficients (PCCs);*

*^3^Genemodel at the soybean genome version 1.0 (http://phytozome.net/soybean);*

*^4^Genemodel at the soybean genome version Wm82.a2.v14 (http://phytozome.net/soybean]);*

*^5^Gene annotation at the Phytozome database (http://phytozome.net/soybean).*

Interestingly, some of the *GmELF4* network genes encode proteins related to abiotic stress responses, such as DnaJ chaperones (Glyma.07G197600 and Glyma.12G130000), chaperonins (Glyma.15G111400), a cold regulated protein (Glyma.17G242100) and a heat shock protein (Glyma.02G288700) (**Table 1**).

## Discussion

### The *ELF4* Putative Genes in Soybean

*ELF4* is a circadian clock gene essential for the maintenance of circadian rhythms and control of flowering in Arabidopsis. A previous study identified five *ELF4*-like genes in Arabidopsis: *ELF4, ELF4-like1, ELF4-like2, ELF4-like3*, and *ELF4-like4* (Khanna et al., 2003). Here we identified a greater number of *ELF4*-like genes in soybean (nine), which agrees with a previous analysis of the soybean genome that shows the presence of complex circadian clock circuitry, generally composed of a greater number of components than that in Arabidopsis (Jung et al., 2012; Marcolino-Gomes et al., 2014; Rodrigues et al., 2015). Interestingly, all the soybean *ELF4* candidates have a DUF1313 motif (IRRV-type) (**Figure 2**), a highly conserved domain exclusively found in plants, characteristic of ELF4 proteins (Li et al., 2015), supporting the function of the soybean candidates to *ELF4* genes/proteins in soybean.

The re-evaluation of a validated RNA-seq library (Rodrigues et al., 2015) allowed us to assess novel data regards the gene expression of the *ELF4* soybean homologes along the day. These data reveals that the mRNA levels of Glyma.18G027500, Glyma.11G229700, and Glyma.14G193700 peak at ZT12 (**Figure 3**), in a similar manner as the Arabidopsis *ELF4* gene (Doyle et al., 2002). This expression profile was validated in Glyma.18G027500 by RT-qPCR in a previous study (Marcolino-Gomes et al., 2014). Interestingly, other *GmELF4* genes showed diverse expression patterns throughout the day (**Figure 3**).

It is known that the accurate oscillation of the transcript levels of the circadian components throughout the day is crucial for accuracy of the circadian rhythms. The Arabidopsis *ELF4* transcripts peak at specific periods allows it to interact with other circadian components to guarantee circadian precision and normal clock function (McWatters et al., 2007; Herrero et al., 2012). Thus, how can the differences found in the mRNA peaks observed for the different *ELF4* genes from soybean be explained? The answer for this question may reside within the evolutionary history of soybean. The current soybean genome is the result of three rounds of polyploidy: (i) a genome triplication before the origin of the rosids (∼130 to 240 million years ago), shared with grape, poplar and Arabidopsis; (ii) a genome duplication early in the legumes (∼58 million years ago); and (iii) a duplication in the *Glycine* lineage (∼13 million years ago) (Severin et al., 2011).

An important feature of duplication is that it produces genetic redundancy, and a duplicated *locus* creates the opportunity for duplicates to explore new evolutionary functions (Flagel and Wendel, 2009). In a previous study of flowering genes in soybean, it was postulated that the differential accumulation of gene copies between soybean and Arabidopsis would be a possible evolutionary innovation that distinguishes the two species from one another (Jung et al., 2012). In addition, it has been verified that, if given sufficient time, one copy of a duplicate pair of genes can accumulate mutations that result in function divergence, or ‘neofunctionalization’ (Flagel and Wendel, 2009). The phylogenetic analyses of Arabidopsis and other angiosperms ELF4 genes reveals a complex gene history of duplication and loss within the ELF4 family (Kolmos et al., 2009). In this context, the different gene expression patterns observed for the *ELF4* genes in soybean may be a reflex of functional divergence due modifications that occurred in this group of genes during the dynamic evolutionary history of the soybean genome. This divergence is possible because the role of sustaining the circadian oscillator, played by *ELF4*, can be fulfilled by the genes that oscillate to peak levels at ZT12 (such as Glyma.11G229700 and Glyma.18G027500), while the other paraloges may assume additional roles in the plant molecular circuitry. Furthermore, this mechanism may contribute to the evolution the circadian clock complexity of plants.

### Functional Characterization *GmELF4* Homologous in Transgenic Arabidopsis

The close phylogenetic relation between Glyma.18G027500 and the Arabidopsis ELF4 major protein (AT2G40080) (**Figure 1**), together with the similar expression patters between these ortholog genes (**Figure 3**) encouraged us to investigate the function of Glyma.18G027500 as a putative *GmELF4* gene. The quantitation of the transcript levels of the putative *GmELF4* in transgenic versus WT plants confirmed that the transgene was highly expressed in line EF7 for both SD and LD, while lines EF14 and EF15 had lower transcript levels (**Figures 4A,C**).

It is known that gene activity is not exclusively determined by the promoter that controls transcription (in our case, the *CaMV35S* promoter): epigenetic mechanisms also influence expression levels by blocking gene transcription or inhibiting mRNA accumulation. Thus, the difference of the transgene expression among lines EF7, EF14, and EF15 (**Figures 4A,C**) can be attributed to several factors including DNA methylation, production of aberrant RNAs, ectopic DNA–DNA interactions and the effect of the *locus* where the transgene was inserted (Stam et al., 1997; Butaye et al., 2005).

The most striking phenotype of the transgenic *GmELF4-ox* lines compared to WT plants was flowering delay. Similarly, McWatters et al. (2007) reported that the constitutive overexpression of the endogenous *ELF4* gene in Arabidopsis resulted in flowering delay under inductive (LD) photoperiods, whereas under non-inductive conditions (SD) plants showed no additional delay in flowering. According to these authors, this phenotype resulted from the role of ELF4 as a floral repressor that coordinates floral transition as part of the photoperiod pathway. Our data reveal that the soybean *ELF4* homologous gene (Glyma.18G027500) is capable of delay Arabidopsis flowering, suggesting the molecular function of *GmELF4* as a flowering repressor. Nonetheless, differently from the *AtELF4* gene, the soybean homolog is also able to produce altered phenotypes (flowering delay) in SD conditions (**Figures 5A,C** and Supplementary Figure 4), indicating that this gene interacts differently with the Arabidopsis flowering control network.

In addition, the transgenic plants also presented a higher number of rosette leaves compared to WT plants (**Figure 6A**). This phenotype is likely a consequence of the flowering *delay* caused by *GmELF4* overexpression, which allowed the plants to invest more in vegetative development, resulting in higher biomass accumulation (**Figure 6B**). Previous analysis of flowering control and biomass yield in *Medicago truncatula* mutants reveals that delayed flowering genotypes produce double the amount of biomass, with less lignin, compared to WT plants, showing that delaying floral initiation could be employed as a convenient biotechnology tool to improve simultaneously biomass quantity and quality in plants (Tadege et al., 2015). In this context, the *GmELF4-ox* phenotypes obtained in this study have great potential for the improvement of crops, such as legume forages, grasses and sugarcane, where flowering delay and improvement of biomass accumulation constitute the main goal.

### *GmELF4* Alters the Expression of Key Arabidopsis Flowering Genes

In Arabidopsis, flowering occurs due the action of the FLOWERING LOCUS T (FT) protein, and the amount of *FT* transcript is directly induced by the transcriptional activator CO protein, which is controlled by the circadian clock and light signaling (Kim et al., 2012, 2013). In turn, the AtELF4 protein is able to bind to the GI protein and target it to subnuclear compartments in the nucleus, sequestering GI from the *CO* promoter, negatively affecting *CO* expression levels and decreasing the amount of *FT* transcript, which results in absence of flower induction (Kim et al., 2012, 2013). Interestingly, our data evidence that the overexpression of *GmELF4* caused a substantial reduction in expression of *AtFT* and *AtCO* (**Figures 4B,D**).

Our phenotypic and molecular data suggest that *GmELF4* may play the role of its homologous gene (*AtELF4*) in reducing the expression of *CO* and *FT*, which results in flowering delay. Further analysis should be carried on to confirm the ability of GmELF4 in sequestering GI from the CO promoter.

A previous study showed that the overexpression of an *ELF4* gene from *Doritaenopsis* Happy Smile × Happy Valentine (*DhEFL4*) alters transgenic Arabidopsis phenotypes by postponing flowering by 12–14 days, indicating that the function of *ELF4* is highly conserved among plants (Chen et al., 2015). Our data shows similar phenotypic results for the *GmELF4* gene, which have not been previously characterized, and our study also provides unpublished data regarding the molecular mechanisms behind these phenotypes.

Structural analysis of the ELF4 protein reveals that it forms a tight homodimer with α-helical composition, critical for the protein function (Kolmos et al., 2009). Thus, studies about the occurrence of this structure in GmELF4 would contribute to a deeper understanding of this protein function. Furthermore, the capacity of the GmELF4 to form dimers could be extended to the formation of a complex with the AtELF4 protein, allowing this complex to act synergistically to generate the delayed flowering phenotypes observed in our transgenic lines.

### The *GmELF4* Network Genes

According to previous studies, the function of every gene is believed to rely on that of another gene(s), and together these genes form complex and intricate networks. Thus, gene co-expression databases are useful resources for predicting gene networks, because co-expressed genes are generally expected to be involved in related cellular functions (Obayashi and Kinoshita, 2009; Obayashi et al., 2009). These analyses show that *GmELF4* network is composed by several homologes of the circadian clock and flowering genes (**Table 1** and Supplementary Figure 5), supporting the participation of *GmELF4* as a component of the soybean circadian clock and flowering genes. Functional studies about the *AtELF4* gene reveals that it functions as a small dimer, and its action is critical for buffering perturbations to the circadian clock evening-loop (Kolmos et al., 2009), a function that may be shared by the *GmELF4* homolog.

Curiously, we also identified many abiotic stress responsive genes composing the *GmELF4* network (**Table 1** and Supplementary Figure 5). Several studies have shown connections between the plant responses to abiotic stresses (e.g., heat, cold, and drought) and the circadian clock (Fowler et al., 2005; Bieniawska et al., 2008; Legnaioli et al., 2009; Wilkins et al., 2010; Marcolino-Gomes et al., 2014; Rodrigues et al., 2015), suggesting a close connection between both pathways. In a previous study, we showed that severe drought stress imposition strongly represses the expression of *GmELF4* in soybean (Marcolino-Gomes et al., 2014). Together, this evidences that *GmELF4* contributes to multiple important plant physiological responses, including the circadian clock, flowering control and responses to abiotic stresses, such as drought. The drought escape strategy entails the completion of the lifecycle in advance of the effects of drought and is a common strategy among annual plants, such as soybean; in *Brassica rapa*, for example, drought escape by early flowering has been proven to be an important adaptive mechanism acquired during plant evolution (Franks, 2011).

Based on our data, we suggest that *GmELF4* may acts as a key negative flowering controller in soybean plants, in the same manner it does in transgenic Arabidopsis plants. Furthermore, its repression in response to drought stress may be part of a mechanism to promote early flowering to escape drought. Additional analyses in transgenic soybean plants (*GmELF4-*ox and/or *elf* mutants) are encouraged to provide additional insights about the flowering control mechanisms in this organism.

## Conclusion

Here, we identified and functionally characterized a negative controller of flowering in soybeans (*GmELF4*) by overexpressing the CDS of this gene in Arabidopsis. The transference of knowledge between model and crop species will contribute to the understanding of the mechanisms underlying several aspects of plant physiology, including flowering. In addition to the contribution to the scientific understanding of the soybean flowering mechanisms, these findings also may contribute to the advance of soybean breeding programs that involve photoperiodic flowering induction, which impacts crop latitudinal and seasonal establishment as well as productivity. In addition, flowering repression phenotypes may be of great interest for crops such as legume forages, grasses, sugarcane and energy cane, where the main feature is biomass accumulation rather than seed production. Altogether, our results may help unveil a new perspective of photoperiodic flowering control in soybean. Future studies are encouraged to confirm the function of *GmELF4* as a molecular hub connecting multiple physiological responses including the circadian clock, flowering control and responses to abiotic stresses.

## Author Contributions

JM-G and AN: contributed to the conception of the work, data acquisition, analysis, and interpretation, drafting the work and final approval of the version to be published; TN, HM, and MB: contributed to data acquisition, analysis, and interpretation as well as manuscript review; LH, RF-P, and FH: contributed to data interpretation and critically revised the manuscript for important intellectual content. All Authors agreed to be accountable for all aspects of the work in ensuring that questions related to the accuracy or integrity of any part of the work were appropriately investigated and resolved.

## Conflict of Interest Statement

The authors declare that the research was conducted in the absence of any commercial or financial relationships that could be construed as a potential conflict of interest.

## Footnotes

1 http://phytozome.net/soybean

2 http://www.ncbi.nlm.nih.gov/protein

3 http://meme-suite.org/tools/meme

4 http://www.ncbi.nlm.nih.gov/geo/query/acc.cgi?acc=GSE69469

5 http://www.geospiza.com/Products/AnalysisEdition.shtml

6 https://imagej.nih.gov/ij/download.html

7 http://www.bioinformatics.nl/cgi-bin/primer3plus/primer3plus.cgi

8 http://www.phytozome.net/search.php?method=Org_Gmax

9 http://http://bioinformatics.cau.edu.cn/SFGD/
