# Supplementary material for: Functional Characterization of a Putative Glycine max ELF4 in Transgenic Arabidopsis and Its Role during Flowering Control
Source: Front Plant Sci. 2017 Apr 20;8:618. doi: 10.3389/fpls.2017.00618 (PMC5397463; doi:10.3389/fpls.2017.00618)
Supplement: Supplementary file 1 [file Image_1.PDF]

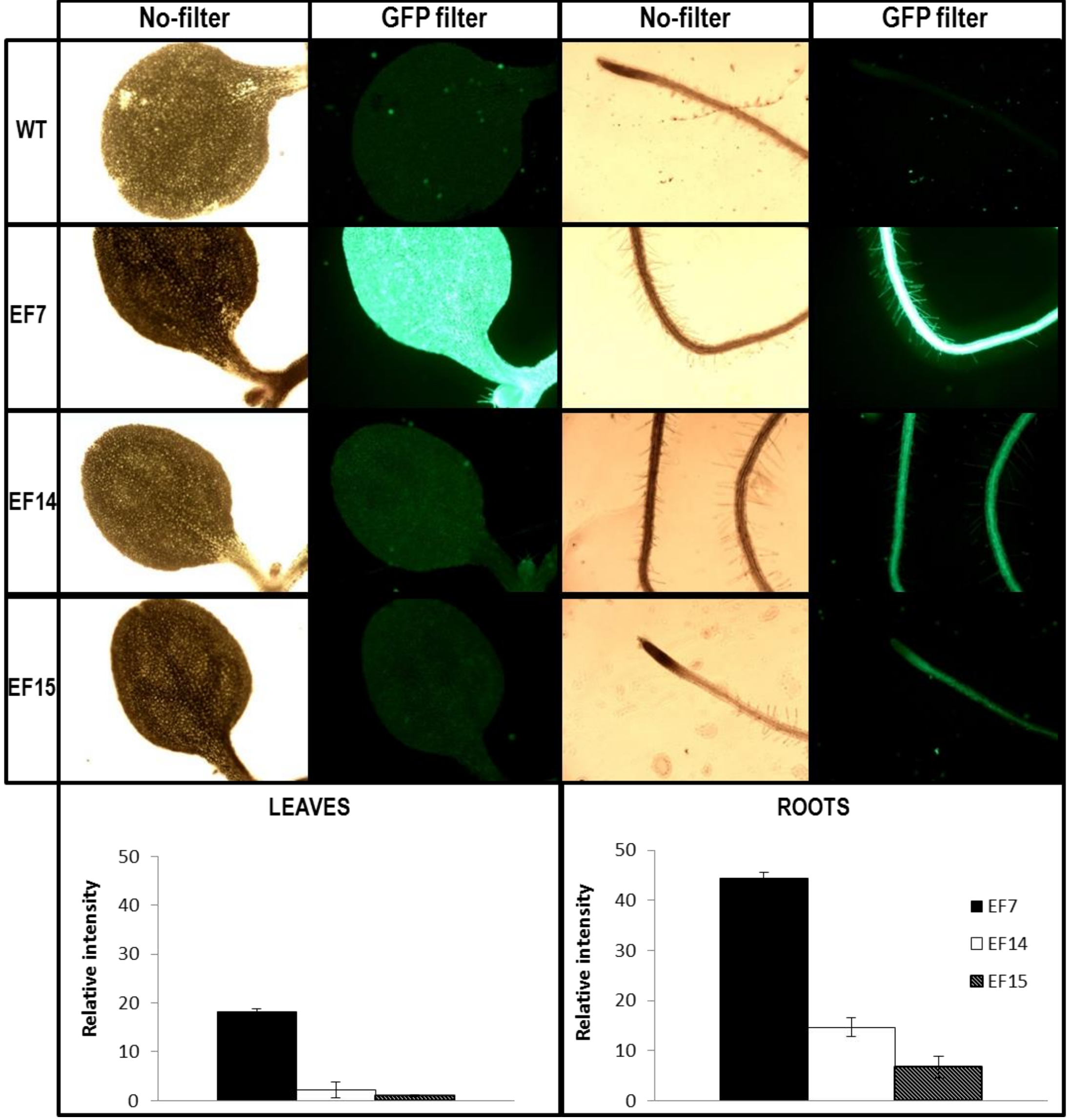


**Supplementary Image 1. GFP expression in leaves and roots of trasngenic Arabidopsis.).** Images show fresh tissues from 5 days-old plants from trasngenic lines (EF7, EF14 and EF15) and wild-type plants analyzed at the optical microscope using commom light (no-filter) and a GFP filter. The GFP quantitation by ImageJ software is presented at the bar-charts where GFP intensity (measured in image mean grey values) of WT plants were used for background normalization of GFP intensity in transgenic plants, generating the relative intensity values; error bars represent standard deviation.
