# Supplementary material for: Functional Characterization of a Putative Glycine max ELF4 in Transgenic Arabidopsis and Its Role during Flowering Control
Source: Front Plant Sci. 2017 Apr 20;8:618. doi: 10.3389/fpls.2017.00618 (PMC5397463; doi:10.3389/fpls.2017.00618)
Supplement: Supplementary file 2 [file Image_2.PDF]

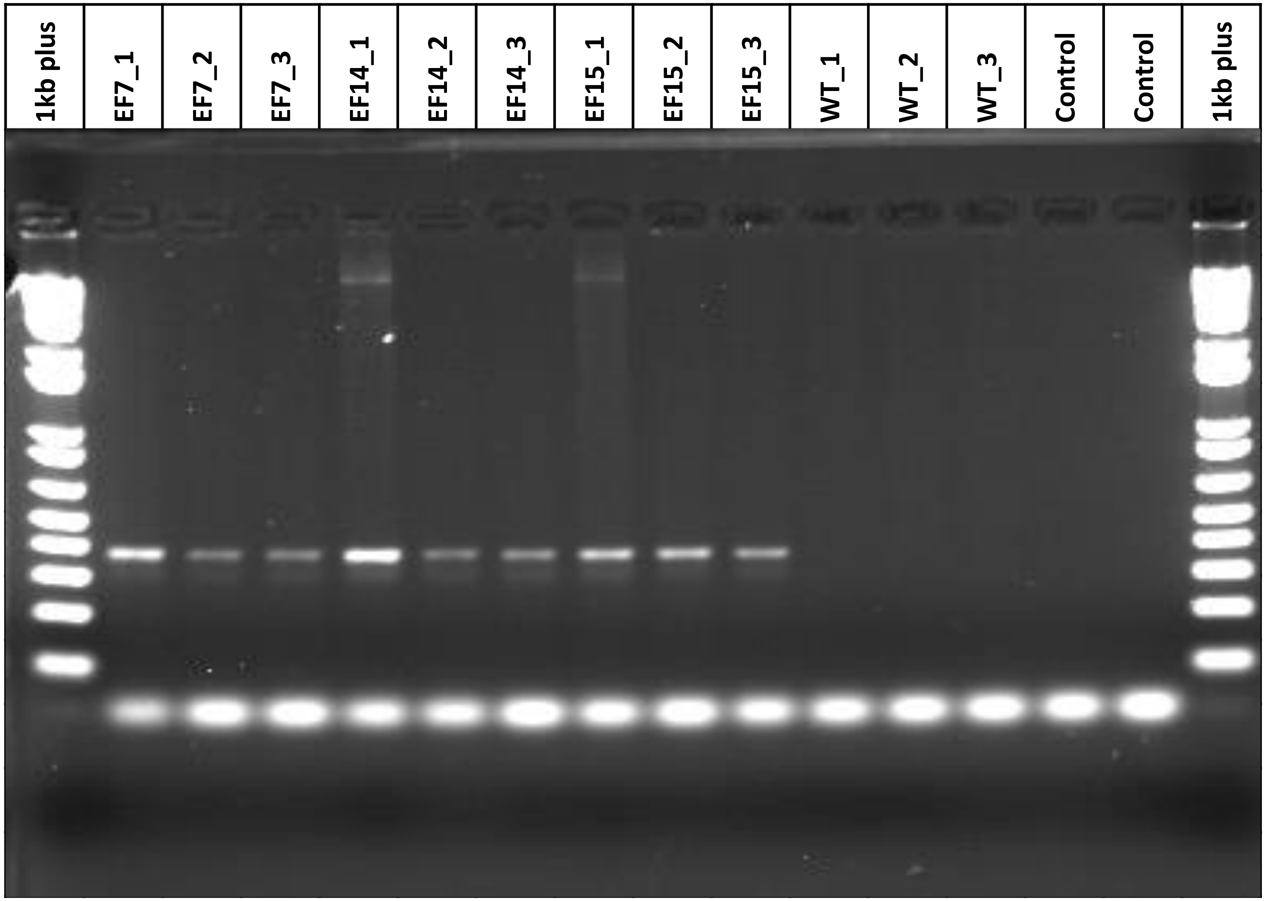

Supplementary Image 2. Transgenic plants confirmation by PCR. Amplification was performed using *GmELF4* forward (5’ TGATTCAGCAGGTGAACGAG 3’) and reverse (5’ GACAACCTTGGAGATGTTGC 3’) primers, generating an amplicon with 385bp. DNA from rosette leaves from three biological replicates (_1, _2, _3) from lines EF7, EF14 and EF15 were used as template. Electrophoresis gel (Agarose 1%) from the PCR products is shown, where lane 1 and 16: 1kb Plus ladder (Invitrogen), lanes 2-4: line EF7, lanes 5-7: EF14; lanes 8-10: EF15; lanes 11-13: wild-type plants; and lanes 14-15: control reactions (no template).
