# Supplementary material for: Functional Characterization of a Putative Glycine max ELF4 in Transgenic Arabidopsis and Its Role during Flowering Control
Source: Front Plant Sci. 2017 Apr 20;8:618. doi: 10.3389/fpls.2017.00618 (PMC5397463; doi:10.3389/fpls.2017.00618)
Supplement: Supplementary file 3 [file Image_3.PDF]

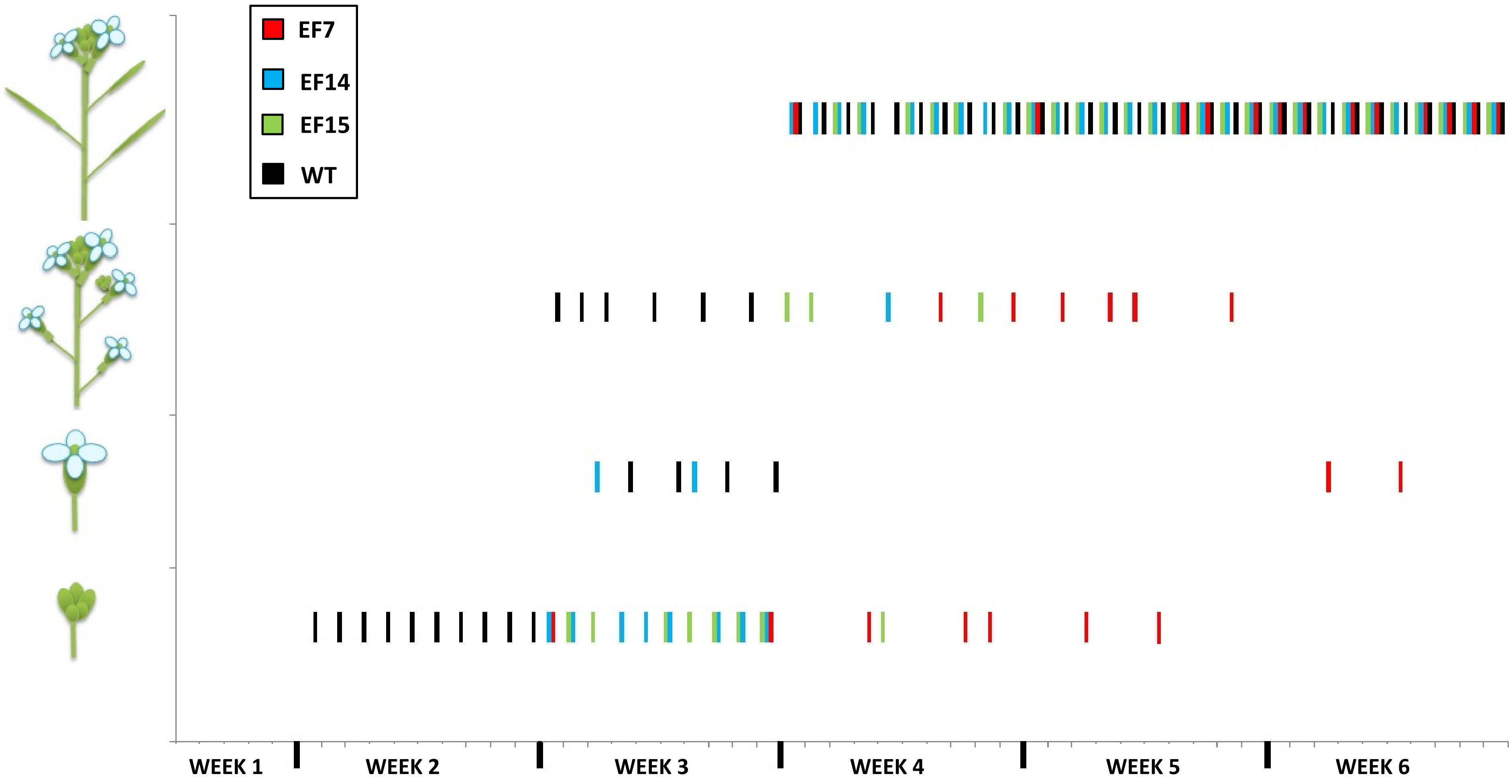


**Supplementary Image 3. Flowering time and progression of silique formation under LD conditions.** Flowering and progression of silique formation from transgenic lines EF7, EF14 and EF15 and from WT plants cultivated under 16h of light are shown. Ten plants (represented by bars) from each genotype were monitored weekly during 6 weeks (abscissa) and were categorized in one of 4 flower/silique stages (vertical axis).
