# Supplementary material for: Functional Characterization of a Putative Glycine max ELF4 in Transgenic Arabidopsis and Its Role during Flowering Control
Source: Front Plant Sci. 2017 Apr 20;8:618. doi: 10.3389/fpls.2017.00618 (PMC5397463; doi:10.3389/fpls.2017.00618)
Supplement: Supplementary file 5 [file Image_5.PDF]

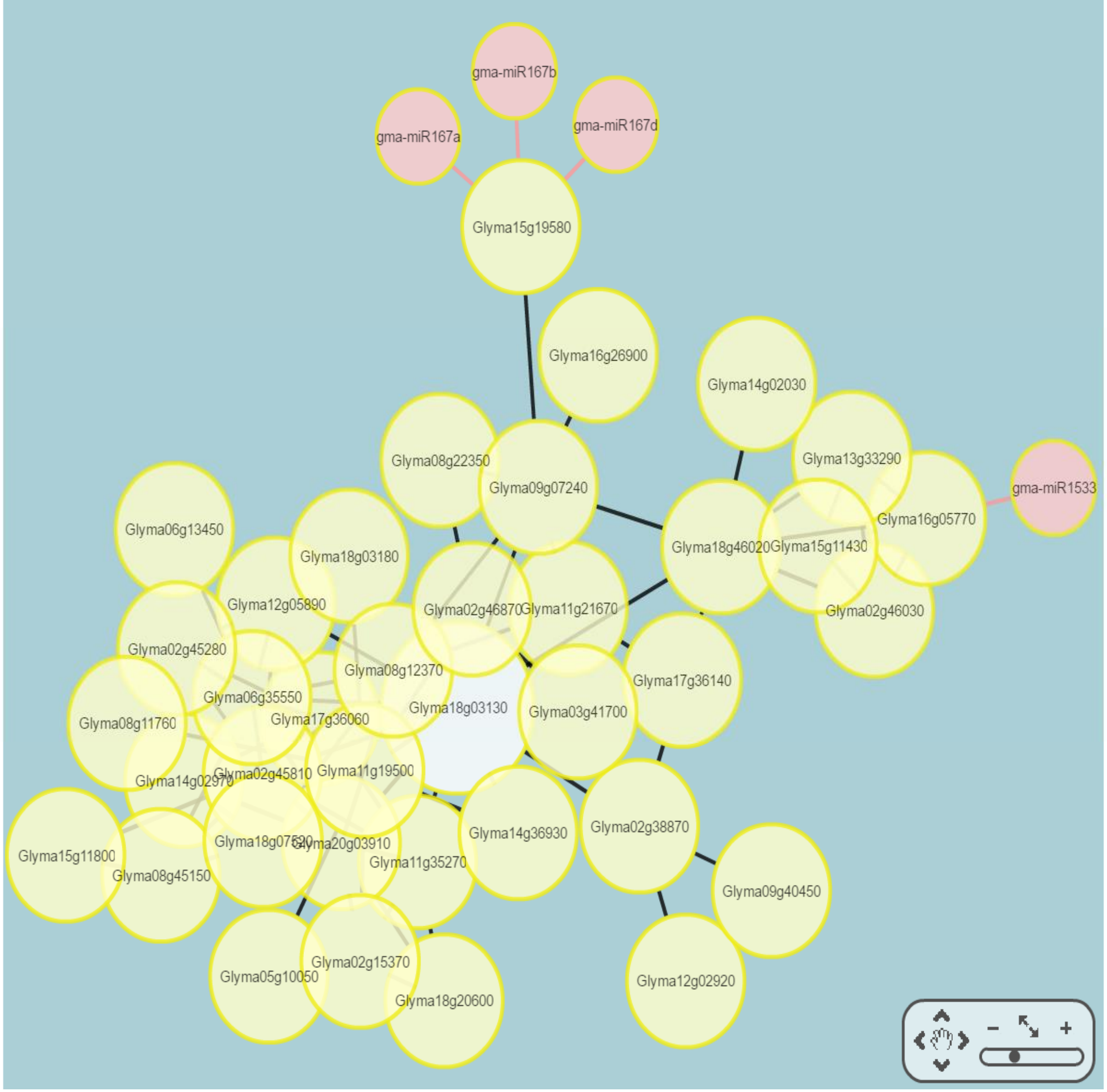

Supplementary Image 5. *GmELF4* network genes. The picture shows *GmELF4*'s co-expression gene regulatory network. Genes are represented for gene models from the soybean genome V1.1. The biggest node is the central gene, surrounded by 41 network genes. **Black** edges represent gene-gene interaction and **pink** represent gene-miRNA interaction. The edge's length corresponds to Mutual rank (MR) values.
